# A Darwinian Plea

**Published:** 1875-04

**Authors:** 


### A DARWINIAN PLEA.

A Darwin philosopher was brought before a justice on a charge of drunkenness. In defence, he said, "Your worship, I am a Darwinian, and I have, I think, discovered the origin of my unfortunate tendency. One of my remotest grandfathers was an anthropoid of a curious turn of mind. One morning, about 4,391,633 B. C., he was looking over his store of cocoanuts, when he picked up one for his breakfast in which the milk had fermented. He drank the liquor and got gloriously drunk, and ever afterwards he always kept his cocoanuts until fermentation took place. Judge, then, whether a tendency handed down through innumerable ancestors, should not be taken in my defence." Casting a sarcastic look at the prisoner, the justice said, "I am sorry that the peculiar arrangement of the atoms of star-dust resulted in giving me a disposition to sentence you to pay a fine of five shillings and costs."

